# Different effects of inspiratory duration and expiratory duration on heart rate deceleration capacity and heart rate asymmetry

**DOI:** 10.1007/s00421-024-05433-2

**Published:** 2024-02-29

**Authors:** Yong-Ping Wang, Terry B. J. Kuo, Guo-Zhi Wang, Cheryl C. H. Yang

**Affiliations:** 1 Department of Anesthesiology, National Taiwan University Hospital, Taipei, Taiwan; 2 Institute of Brain Science, National Yang Ming Chiao Tung University, Taipei, Taiwan; 3 Sleep Research Center and Institute of Brain Science, National Yang Ming Chiao Tung University, No. 155, Sec. 2, Li-Nong St., Taipei, 11221 Taiwan; 4 Brain Research Center, National Yang Ming Chiao Tung University, Taipei, Taiwan; 5 Department of Education and Research, Taipei City Hospital, Taipei, Taiwan; 6 Clinical Research Center, Taoyuan Psychiatric Center, Ministry of Health and Welfare, Taoyuan, Taiwan; 7 Department of Surgery, Chung Shan Medical University Hospital, Taichung, Taiwan

**Keywords:** Acceleration, Deceleration, Expiration, Heart rate, Inspiration

## Abstract

**Purpose:**

Low values of heart rate deceleration capacity (DC) and heart rate asymmetry (HRA) are associated with cardiovascular risks. Slow respiration has been proven to enhance the magnitudes of these indexes, but individual inspiratory (T_I_) and expiratory (T_E_) durations were not controlled in most studies. This study aims to examine whether the effects of T_I_ and T_E_ on these indexes would be the same and, if not, how to adjust T_I_ and T_E_ to maximize the effect of slow respiration.

**Methods:**

We evaluated 14 seated healthy young adults who randomly controlled their breathing to nine combinations of T_I_ and T_E_, each chosen respectively from 2, 4, and 6 s. A 5-min R-R interval time series was obtained from each study period for further analysis.

**Results:**

The magnitude of DC increased when T_I_ or T_E_ increased, while that of acceleration capacity (AC) remained almost unchanged by T_I_. We further defined a new index as 100 × DC^2^/(DC^2^ + AC^2^) and found it to be correlated with conventional Guzik’s (*r* = 0.94) and Porta's (*r* = 0.99) indexes of HRA during different combinations of T_I_ and T_E_. Increasing T_I_ and increasing T_E_ both enhanced the magnitudes of HRA indexes, with T_I_ taking effect when ≤ 4 s, and T_E_ taking effect when > 4 s. DC and HRA indexes were maximized with a T_I_ of 4 s and a T_E_ of 6 s.

**Conclusion:**

We suggest that a T_I_ of 3–4 s with a T_E_ of 7–6 s is an appropriate standard for slow respiration.

**Supplementary Information:**

The online version contains supplementary material available at 10.1007/s00421-024-05433-2.

## Introduction

Heart rate variability (HRV) has been widely used for assessing cardiovascular risk (TaskForce 1996). Traditional time- and frequency-domain analytic methods typically focus on the overall magnitude of variations. However, the phase-rectified signal averaging (PRSA) technique (Bauer et al. 2006b) allowed the evaluation of heart rate accelerations and decelerations separately. Studies have shown that low magnitudes of deceleration capacity (DC) are more strongly associated with mortality than those of acceleration capacity (AC) in patients after myocardial infarction (Bauer et al. 2006a).

On the other hand, the Poincaré plot analysis enables a direct comparison of heart rate accelerations and decelerations. Notably, in a significant percentage of resting healthy individuals, the contributions of decelerations to short-term variance are greater than those of accelerations (Guzik et al. 2006), while heart rate accelerations consist of more beats than heart rate decelerations (Porta et al. 2008). Consequently, two indexes of heart rate asymmetry (HRA) have been proposed. Guzik’s index (HRA_GI_) and Porta’s index (HRA_PI_) are associated with a sense of well-being when their values are larger than 50% (symmetry). Conversely, studies have reported lower values of HRA in healthy individuals experiencing acute mental stress (Visnovcova et al. 2014), negative emotions (Kaczmarek et al. 2019), or aging (Costa et al. 2005), as well as in patients with diabetes mellitus (Guzik et al. 2010), heart failure (Costa et al. 2005), myocardial infarction (Guzik et al. 2012), or major depression (Tonhajzerova et al. 2012).

Respiration is a major rhythmic process that influences HRV. A study shows that both DC and HRA_GI_ increase at lower respiratory rates (Wang et al. 2015). Consequently, enhancing the magnitudes of DC and HRA through slow respiration may prove beneficial for patients recovering from myocardial infarction. Conversely, a high respiratory rate has been associated with a high mortality risk (Barthel et al. 2013; Dommasch et al. 2014). The average human respiratory rate ranges from 12 to 22 breaths per minute (Tobin et al. 1983). Notably, paced breathing at 6 breaths per minute (10 s per breath) is a well-known technique that improves cardiorespiratory performance (Bernardi et al. 1998; Raupach et al. 2008; Joseph et al. 2005). Moreover, slowing the respiratory rate to below 10 breaths per minute (over 6 s per breath) has also been used as a treatment of hypertension (Sharma et al. 2011).

However, most studies in this area did not control for individual inspiratory duration (T_I_) and expiratory duration (T_E_). Therefore, we sought to investigate whether the effects of T_I_ and T_E_ on the magnitude of DC or HRA are the same, and if not, how adjustments in T_I_ and/or T_E_ could maximize the effect of slow respiration. For this study, participants were asked to control their breathing with nine combinations of T_I_ and T_E_, each chosen from 2, 4, and 6 s. Beat-to-beat heart period variations in the nine study sections were analyzed. To depict the relation between DC, AC, and HRA, we further defined a new index as 100 × DC^2^/(DC^2^ + AC^2^). The primary aim of this study is to compare the effects of T_I_ and T_E_ on these autonomic indexes.

## Methods

### Participants

We studied young healthy volunteers with normal ECG readings. They were nonsmokers and had no history of systemic medical problems or medication intake. All individuals abstained from caffeine and alcohol intake for 12 h before the tests. This study was approved by the Institutional Review Board of National Yang-Ming University (YM104060E-1). All participants provided their written informed consent.

### Experimental protocol

The experiment was conducted in a quiet room with a room temperature of 23–25 °C. Participants were seated and instructed to control their breathing as per acoustic and visual signals. We developed a computer program to generate high- and low-pitch beeps alternatively, with intervals based on preset durations. Additionally, a vertical tube containing a colored fluid was displayed on the screen, providing further cues. Participants were instructed to breathe in (and out) when they heard high-pitched (and low-pitched) beeps and saw the fluid level rising (and falling). They adjusted the tidal volume to a comfortable level on their own. The durations of inspiration and expiration were chosen from 2, 4, and 6 s, resulting in nine possible combinations (I2-E4, I6-E2, and so on.). These nine combinations were randomly arranged and executed one after another. In each session, respiration was controlled for 7 min after a 3-min resting time.

### Measurements and basic analysis

Respiratory movement was detected using an elastic belt secured around the participant’s chest at the level of the xiphoid process. For ten of the 14 participants, an additional elastic belt was secured around the abdomen. Continuous ECG readings and measurements of thoracic and abdominal circumferences were taken using polysomnography equipment (Embla, Inc., Broomfield, CO, USA). The waveform data set was saved simultaneously after digital conversion at a sampling rate of 500 Hz (for ECG) or 200 Hz (for respiratory movement).

**Fig. 1:**
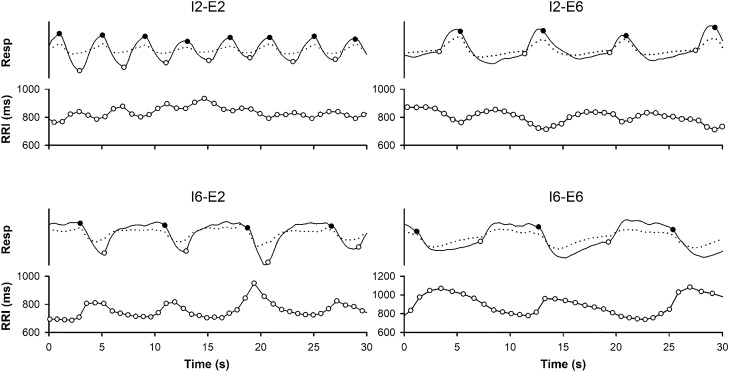
Representative examples of the recordings of respiratory signals and simultaneous beat-to-beat R-R interval time series. Respiratory signals (Resp) were detected using two elastic belts secured around the participant’s abdomen (dotted line) and the chest at the level of the xiphoid process (solid line) during paced breathing under different inspiratory and expiratory durations (I2–E2 et al*.*). The occurrences of inspiration onset (open circle) and expiration onset (solid circle) are marked

The original recordings were analyzed using a commercial software package (MATLAB for Windows version 4.2) and associated computer programs written by us. The onset times of inspiration and expiration were manually identified from respiratory waveforms based on the relationship between beep signals and the changes in the respiratory waveforms (Fig. 1). Participants were excluded if identification was difficult due to the low quality of the respiratory waveforms.

**Fig. 2:**
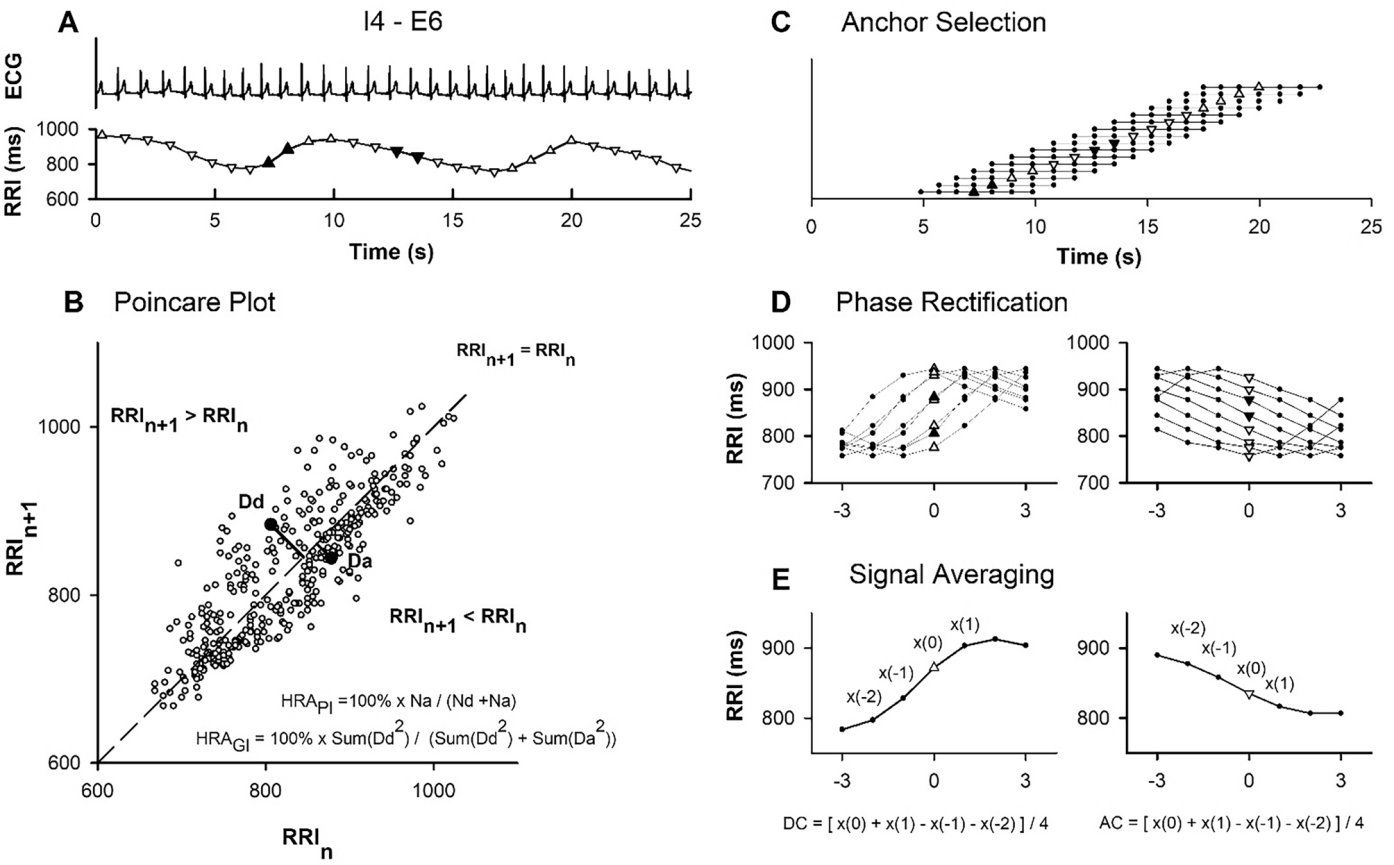
Simplified presentation for the Poincaré plot analysis and phase-rectified signal averaging (PRSA) technique. A, Representative example of RRI time series derived from ECG recording during paced breathing when inspiratory and expiratory durations were set at 4 and 6 s, respectively. Acceleration and deceleration anchor points characterized by a shorter or longer RRI value than the preceding value are marked by downward and upward open triangles, respectively. Benchmarks (solid triangle) are used for further presentation. B, Presentation for the Poincaré plot analysis of RRI time series. Nd and Na are the numbers of deceleration (RRIn + 1 > RRIn) and acceleration (RRIn + 1 < RRIn) points, respectively. Dd and Da are the distances of deceleration and acceleration points from the line of identity (RRIn + 1 = RRIn), respectively. C–E, Simplified presentation for the PRSA technique (see Methods)

The temporal positions of all R-wave peaks in the ECG waveform were detected (Wang et al. 2013). A visually stable 300-s section of R-R interval (RRI) time series was selected from the 7-min original data. The time at which each RRI occurred was set to be 0.3 s after the occurrence of the first R-wave peak (Fig. 2A), which approximates the occurrence of corresponding systolic peak in arterial blood pressure. The stationary nature of the RRI time series was examined by dividing the section into 10 segments of equal length and applying reverse arrangement tests at the 5% significance level to the segment means and variances. A section that did not pass both tests was excluded. Nine sections with different breathing protocols were available for each participant. Participants were excluded if more than two sections were excluded.

### Spectral analysis

The magnitude of respiratory sinus arrhythmia, a form of respiration-related HRV, was assessed by measuring the square root of the spectral power in the respiratory frequency range (RF; ± 0.05 Hz centered on each paced breathing frequency). Spectral analysis was carried out using Welch’s averaged, modified periodogram method (MATLAB Signal Processing Toolbox version 3.0). In brief, beat-to-beat RRI data were interpolated using the cubic spline method and resampled equidistantly at a rate of 4 Hz. A 256-s section, consisting of 1024-point values, was divided into three 512-point segments that overlapped by half. A fast Fourier transform algorithm was applied to each segment after linear detrending and the application of a Hanning window. The averaged power spectral density curve of the three segments was computed.

### PRSA technique

Acceleration- and deceleration-related RRI variations were assessed separately using the PRSA technique (Bauer et al. 2006b). In brief, the RRI time series was scanned to identify acceleration and deceleration anchor points characterized by a shorter or longer value than the preceding value, respectively (Fig. 2A). Once an anchor was determined, a seven-point segment, including the anchor point, the interval immediately following the anchor, and two intervals immediately preceding the anchor, was selected (Fig. 2C). Because anchor points are usually close to one another, these overlapping segments within the RRI time series were aligned at the anchors (Fig. 2D) and then averaged (Fig. 2E). Acceleration capacity (AC) and deceleration capacity (DC) were calculated as a quarter of the difference between two sums. Specifically, it involved the sum of the averaged anchor interval with the next averaged interval and the sum of the two averaged intervals preceding the anchor point. In this study, we further defined 100 × DC^2^/(DC^2^ + AC^2^) as an index of HRA (HRA_DC_).

### Poincaré plot analysis

HRA was assessed by plotting consecutive points of the beat-to-beat RRI time series on a two-dimensional plane, commonly known as Poincaré plot (Fig. 2B). All points on the line of identity, which passes through the origin with a 45° slope, have equal consecutive RRIs. Any point above or below the line of identity corresponds to an increasing or decreasing RRI, respectively. Guzik’s index of HRA (HRA_GI_) was defined as the percentage of the sum of squared distances of deceleration points to the sum of squared distances of all acceleration and deceleration points from the line of identity (Guzik et al. 2006). On the other hand, Porta’s index of HRA (HRA_PI_) was defined as the percentage of the number of acceleration points with respect to the number of acceleration and deceleration points (Porta et al. 2008).

### Statistical analysis

Based on Shapiro–Wilk normality tests, one-way repeated-measures analysis of variance, or Friedman repeated-measures analysis of variance on ranks, was performed to evaluate the differences across various respiratory settings. Paired *t* test was used if appropriate. Two-way repeated-measures analysis of variance was used to analyze the combined effects of inspiratory and expiratory settings. Post hoc pairwise multiple comparisons were conducted using Tukey’s test where necessary. A 95% confidence interval was used to detect the differences between the magnitudes of AC and DC and the differences of HRA indexes from 50% (symmetry). Within-subject Pearson correlation analysis (Bland and Altman 1995) was used to evaluate the relationship among various indexes of HRA. Statistical significance was indicated if *P* < 0.05.

## Results

Fourteen participants (6 men and 8 women) with an average age of 23 ± 2 years were included in the study. The participants exercised for an average of 3.4 ± 3.1 h/week. Their mean body height, body weight, and body mass index were 166 ± 10 cm, 61 ± 15 kg, and 21.8 ± 3.7 kg/m^2^, respectively. Ten participants had complete data for all nine sections. Three participants had one section, and one participant had two sections excluded from the analysis. Six sections had complete data from all 14 participants, one section (I4–E6) had data from 13 participants, and two sections (I2–E4 and I6–E6) had data from 12 participants.

**Fig. 3:**
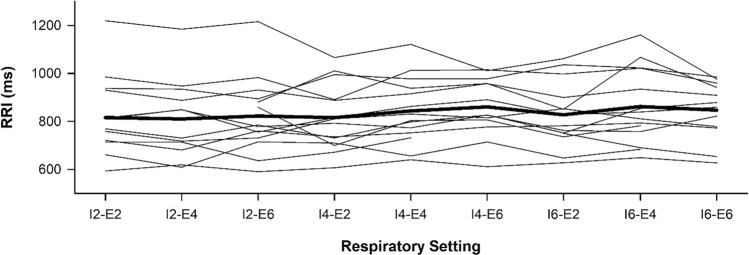
The average RRI is comparable at various respiratory settings. The average RRI changes non-significantly during paced breathing with different settings for inspiratory (I2, I4, and I6) and expiratory (E2, E4, and E6) durations. Individual data (*n* = 14, thin line) and mean values (thick line) are presented. Friedman repeated-measures analysis of variance on ranks was used to analyze differences across various respiratory settings (Chi-square = 19.65, *P* = 0.012). But difference was not observed in all pairwise multiple comparison procedures (Tukey’ test)

**Fig. 4:**
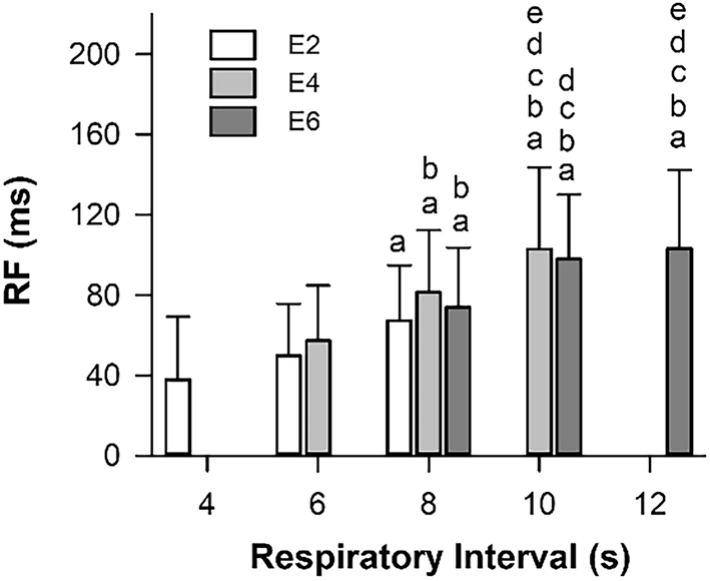
The magnitude of respiration-related HRV increases with respiratory interval. The square root of the spectral power of HRV in the respiratory frequency range (RF) increases progressively as the respiratory interval increases during paced breathing with different settings for inspiratory (I2, I4, and I6) and expiratory (E2, E4, and E6) durations. Values are presented as mean ± standard deviation (*n* = 14) and were analyzed using one-way repeated-measures analysis of variance (*F* = 20.7, *P* < 0.001). Tukey’s test was used for multiple comparisons. Here, a, b, c, d, and e indicate difference from I2E2 (4 s), I4E2 (6 s), I2E4 (6 s), I6E2 (8 s), and I2E6 (8 s), respectively

In all sections, average breath-to-breath respiratory durations were close to values that were expected and significantly differed in inspiratory and expiratory settings (Supplementary material online Table S1). The average RRI was comparable at various respiratory settings (Fig. 3), while the magnitude of respiratory sinus arrhythmia increased with the respiratory interval (Fig. 4).

**Fig. 5:**
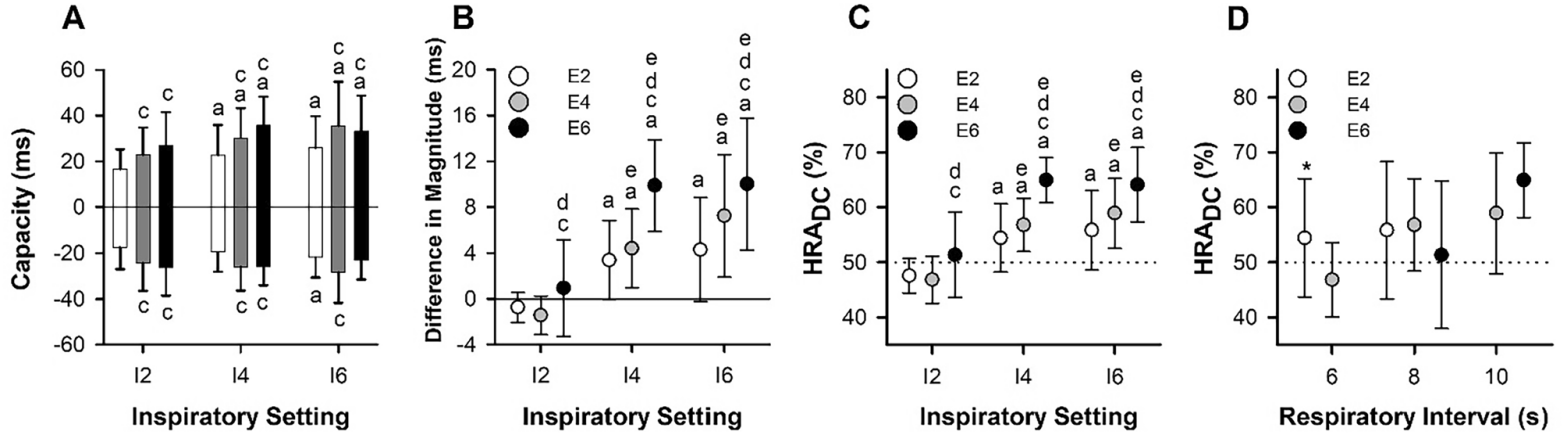
Effects of respiratory phase durations on the deceleration capacity and acceleration capacity of heart rate. **A**–**C,** Effects of inspiratory (I2, I4, and I6) and expiratory (E2, E4, and E6) durations on the DC (positive bars) and AC (negative bars) of heart rate (**A**), their difference in magnitude (DC − AC) (**B**), and the data for HRA_DC_, which was defined as 100 × DC^2^/(DC^2^ + AC^2^) (**C**). Values are mean ± standard deviation (**A**) or 95% confidence interval (*n* = 14). Two-way repeated-measures analysis of variance was performed to analyze the effects of inspiratory and expiratory durations. F and *P* values are presented in the Supplementary material online Table S2. Tukey’s test was used for multiple comparisons. Here, a, b, c, and d indicate difference from I2, I4, E2, and E4, respectively, and e indicates difference from 0 (**B**) or 50% (**C**). **D,** Effect of respiratory time ratio on the HRA_DC_ during paced breathing with the same respiratory interval. Values are mean ± standard deviation (*n* = 14). Paired t-test or one-way repeated-measures analysis of variance was performed. *indicates difference between I4-E2 and I2-E4

Regarding the DC, there was a significant increase from I2 to I4 (*F* = 7.87, *P* = 0.002) and from E2 to E4 (*F* = 12.46, *P* < 0.001). However, further increase from I4 to I6 or from E4 to E6 was nonsignificant, and there was no significant interaction between T_I_ and T_E_ (*F* = 1.03, *P* = 0.40). Notably, the magnitude of DC reduced slightly during I6-E6 (Fig. 5A, positive bars). On the other hand, the magnitude of AC showed little correlation with T_I_ (*F* = 0.38, *P* = 0.69). It increased from E2 to E4 (*F* = 9.12, *P* < 0.001), but no change or even a decrease (I6-E6) was observed from E4 to E6. A significant interaction between T_I_ and T_E_ was found (*F* = 3.57, *P* = 0. 01). (Fig. 5A, negative bars).

Comparing the magnitudes of DC and AC during different inspiratory and expiratory settings (Fig. 5B), we observed that DC was smaller than AC during I2-E2 and I2-E4. However, DC became larger than AC when both T_I_ and T_E_ were ≥ 4 s. Correspondingly, HRA_DC_ increased to > 50% at the same time (Fig. 5C). HRA_DC_ significantly increased from I2 to I4 (*F* = 10.61, *P* < 0. 001), but no further increase was observed between I4 and I6. On the other hand, there was no significant difference in HRA_DC_ between E2 and E4, but it increased significantly from E4 to E6 (*F* = 10.02, *P* < 0. 001). There was no significant interaction between T_I_ and T_E_ for HRA_DC_ (*F* = 0.92, *P* = 0.46). The magnitude of HRA_DC_ peaked during I4-E6 and I6-E6. There was no difference between them (*t* = − 0.73 with 10 degrees of freedom, two-tailed *P* = 0.49).

We further evaluated the effect of I/E. HRA_DC_ during I4-E2 breathing was significantly larger than that of I2-E4 (*t* = − 2.46 with 11 degrees of freedom, *P* = 0.03). However, no difference was observed when the respiratory interval increased to 8 s (*F* = 0.89, *P* = 0.42) or 10 s (*t* = 1.46 with 12 degrees of freedom, two-tailed *P* = 0.17) (Fig. 5D).

**Fig. 6:**
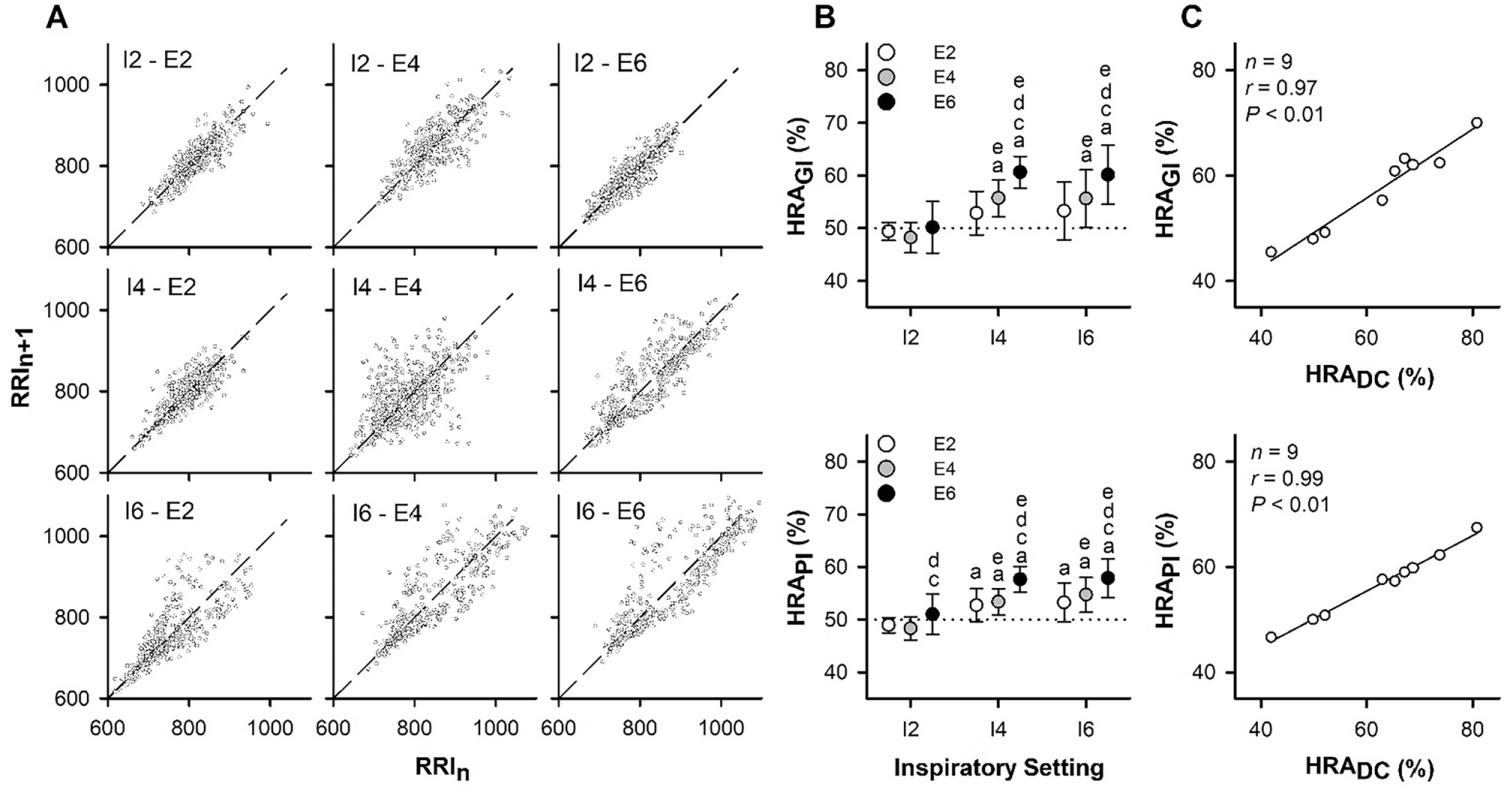
Effects of respiratory phase durations on the conventional indexes of heart rate asymmetry. **A,** Representative examples of Poincaré plots of RRI time series recorded from a participant at nine combinations of inspiratory and expiratory durations of 2, 4, or 6 s each. **B,** Effects of inspiratory (I2, I4, and I6) and expiratory (E2, E4, and E6) durations on the HRA_GI_ and HRA_PI_. Values are mean ± 95% confidence interval (*n* = 14). Two-way repeated-measures analysis of variance was performed to analyze the effects of inspiratory and expiratory durations. F and *P* values are presented in the Supplementary material online Table S2. Tukey’s test was used for multiple comparisons. Here, a, c, and d indicate difference from I2, E2, and E4, respectively, and e indicates difference from 50%. **C,** Representative example of within-subject relationship between HRA_DC_ and HRA_GI_ or HRA_PI_. Values were obtained from a participant under different settings for inspiratory and expiratory durations

Poincaré plots revealed altered patterns of beat-to-beat RRI variations due to changes in respiratory durations (Fig. 6A). The effects of T_I_ and T_E_ on HRA indexes (HRA_GI_ and HRA_PI_) (Fig. 6B) were found to be similar to those observed on HRA_DC_ (Fig. 5C). Remarkably, HRA_DC_ exhibited a strong correlation with the HRA_PI_ (*n* = 121, *r* = 0.994, *P* < 0.001) and HRA_GI_ (*n* = 121, *r* = 0.942, *P* < 0.001). Figure 6C presents representative examples of within-subject relationships in a participant under different T_I_ and T_E_ settings.

## Discussion

The main findings of this study demonstrated that T_I_ and T_E_ had distinct effects on the magnitudes of DC and AC. The newly defined index (HRA_DC_) quantifies the difference between them and showed a close correlation with the conventional HRA_GI_ and HRA_PI_. Both increasing T_I_ and increasing T_E_ had a positive impact on the magnitudes of the HRA indexes. However, their effects differed in terms of timing. T_I_ had a significant effect when it was ≤ 4 s, whereas T_E_ showed its influence when > 4 s. As a result, the optimal combination for maximizing DC and the HRA indexes was found to be a T_I_ of 4 s and a T_E_ of 6 s. These findings shed light on the specific roles of T_I_ and T_E_ in modulating heart rate variability and asymmetry, providing valuable insights for the optimization of respiratory settings in relation to cardiac autonomic regulation.

In our study, the observed changes in average RRI and respiratory sinus arrhythmia with varying respiratory interval were consistent with previous reports (Brown et al. 1993; Angelone and Coulter 1964). Interestingly, the magnitude of AC was found to be almost unaffected by T_I_, contrasting with DC that showed significant changes with T_I_ (Fig. 5A). This observation aligns with previous findings when investigating the effects of isolated deep inspirations, where slow and fast inspirations produced identical magnitudes of acceleratory heart rate responses but different deceleration responses, with slow inspirations resulting in larger deceleration responses than fast inspirations (Stern and Anschel 1968). As a result, T_I_ emerged as a major factor contributing to the difference between the magnitudes of DC and AC (Fig. 5B). Conversely, the magnitudes of both DC and AC were enhanced with increased T_E_. The change in the magnitude of DC was relatively larger than that of AC, particularly at T_E_ of 6 s (Fig. 5A). Hence, T_E_ also played a role in the difference between the magnitudes of DC and AC (Fig. 5B). Notably, the distinct effects of T_I_ and T_E_ on the magnitudes of DC and AC, contributing to the overall asymmetry, were consistent with their effects on HRA_DC_ (Fig. 5C).

In our study, we replicated the computation method of HRA_GI_ and introduced a novel index, HRA_DC_, defined as 100 × DC^2^/ (DC^2^ + AC^2^). DC and AC were computed using the formula (RRIn + 1 + RRIn − RRIn-1 − RRIn-2)/4, where RRIn represents the anchor point. In the context of the Poincaré plot, the squared distance of a point (RRIn-1, RRIn) from the line of identity is given by (RRIn − RRIn-1)^2^/2. Therefore, we squared DC and AC values to calculate HRA_DC_. Interestingly, HRA_DC_ exhibited a strong correlation with HRA_PI_ and HRA_GI_ (Fig. 6C). The impacts of T_I_ and T_E_ on HRA_DC_ (Fig. 5C) mirrored those observed for HRA_GI_ and HRA_PI_ (Fig. 6B). Consequently, this new index served as a bridge connecting the PRSA technique and Poincaré plot analysis. We used it to show that common autonomic indicators obtained by these methods are closely related in some fixed form.

The effects of respiratory interval on HRA have been previously reported, with HRA increasing as the respiratory interval lengthens (Wang et al. 2013, 2015). The impact of I/E on HRA depends on the specific respiratory interval. For instance, prior research indicates that during 4-s breathing, HRA is greater at 1:1 (T_I_ = 2 s) than at 1:2 (T_I_ = 1.3 s) and 1:3 (T_I_ = 1 s), but no difference is observed among 1:1 (T_I_ = 5 s), 1:2 (T_I_ = 3.3 s) and 1:3 (T_I_ = 2.5 s) during 10-s breathing (Wang et al. 2013). Similarly, HRA significantly increases at 2:1 (T_I_ = 3 s) and 1:1 (T_I_ = 2.25 s) compared to 1:2 (T_I_ = 1.5 s) during 4.5-s breathing (Klintworth et al. 2012). Our current findings align with these prior studies. Interestingly, HRA_DC_ at 2:1 (T_I_ = 4 s) is significantly greater than at 1:2 (T_I_ = 2 s) during 6-s breathing, while HRA_DC_ remains unaffected by I/E during 8- and 10-s breathing (Fig. 5D). Taken together, these observations emphasize the pivotal role of T_I_, especially at smaller values. In our investigation, we observed that the effect of T_I_ is maximized at 4 s. Notably, we propose that 2.5 s may represent a critical point since HRA does not exceed 50% even at I2-E6 (8-s breathing; Fig. 5C), yet it becomes significantly greater than 50% at I2.5-E2.5 (5-s breathing) (Wang et al. 2015).

Conversely, T_E_ seems to play a more significant role at larger values. For instance, HRA at I2.5-E2.5 is smaller than at I5-E5 (Wang et al. 2015), whereas HRA at I2.5-E7.5 is comparable to that at I5-E5 (Wang et al. 2013). Our data also indicate that the magnitude of HRA is maximized with a T_E_ of 6 s. Collectively, these results suggest that an optimal approach to maximize the effect of slow respiration would involve a T_I_ of 3–4 s paired with a T_E_ of 7–6 s. Previous studies have applied paced breathing with a T_I_ of 3 s and a T_E_ of 7 s (Raupach et al. 2008; Tsai et al. 2015), which is in line with our proposed optimal combination.

The underlying mechanism of HRA appeared to be closely linked to cardiac vagal activity, given that both DC and AC have been demonstrated to rely solely on vagal activity in a model study (Pan et al. 2016). Further support can be drawn from physiological findings. Respiration is known to mechanically induce fluctuations in intrathoracic pressure, cardiac filling, and arterial pressure, which subsequently triggers baroreceptor reflex responses leading to variations of RRI in accordance with the respiratory cycle (Zhang et al. 2002). The cardiac baroreflex response is characterized by asymmetry; the slopes of RRI lengthening during arterial pressure increase are steeper than the slopes of RRI shortening during arterial pressure decrease. Interestingly, changes in muscle sympathetic nerve activity exhibit comparable patterns during both falling and rising arterial pressure (Rudas et al. 1999). Similar RRI responses are observed with the application of the neck chamber technique, which triggers baroreceptor activity (Eckberg 1980). Notably, atropine abolishes RRI responses to both neck suction and neck pressure, while propranolol only augments RRI prolongation, leaving RRI shortening unaffected (Eckberg 1980).

It is important to note that mean cardiac vagal activity might remain relatively unchanged during varying respiratory phase durations. Mean cardiac vagal tone, as estimated from the mean RRI under β-adrenergic blockade, has been found to be unaffected by changes in respiratory rate or tidal volume (Grossman et al. 1991; Hayano et al. 1994). In line with this, the mean RRI in our study, although not under β-adrenergic blockade, remained comparable across the different respiratory settings. However, the heightened HRA magnitude observed during slow respiration implies a periodic short-term and high-intensity activation of vagal activity coupled with a gradual vagal withdrawal. The alterations in vagal activity align with clinical observations that carry significant implication. The reduction in DC that corresponds to the decreases in vagal activity has been identified as a marker associated with increased mortality in patients who have experienced an acute myocardial infarction. Moreover, DC, compared to AC, is a more accurate and sensitive parameter for risk prediction (Bauer et al. 2006a).

In this study, we investigated young healthy volunteers in the sitting position. The results may be different in subjects with different posture or with an altered baroreflex function such as elderly, diabetic, or hypertensive individuals. Inspiratory and expiratory durations were controlled at 2, 4, and 6 s to cover the physiological ranges in breathing frequency and respiratory time ratio. The reverse ratio was also tested here. Whether the results would be the same at shorter or longer respiratory durations was not evaluated. The speed of inhalation and exhalation was not controlled in this study. We asked the participants to adjust tidal volume by themselves in order to maintain constant alveolar ventilation and normal arterial CO_2_ levels. Therefore, our results should be considered as the combined effects of respiratory duration and tidal volume. There were no training sessions before the 7-min formal recordings, but 2-min data were allowed to be excluded if visually unstable. We further examined the stationary nature of selected RRI time series using reverse arrangement test. Participants were excluded if necessary.

In conclusion, our study delved into the intricate interplay between respiratory phase durations and heart rate variations. The findings underscore the distinct impacts of T_I_ and T_E_ on both DC and HRA. Notably, while a T_I_ exceeding 4 s did not yield significant benefit, a T_E_ surpassing 4 s exhibited a positive effect in maximizing the influence of slow respiration on these autonomic indexes. Based on our results, we propose that a targeted T_I_ of 3 to 4 s coupled with a T_E_ of 7 to 6 s constitutes an optimal standard for achieving the desired outcomes of slow respiration. This recommendation is intended to guide the practice of controlled breathing techniques for individuals seeking to modulate heart rate variability and enhance autonomic balance.

### Supplementary Information

Below is the link to the electronic supplementary material.

Supplementary file1 (DOCX 26 KB)

## Data Availability

The data that support the findings of this study are available from the corresponding author upon reasonable request.

## Abbreviations

AC: Acceleration capacity
DC: Deceleration capacity
ECG: Electrocardiography
HRA_GI_: Guzik’s index of heart rate asymmetry
HRA_PI_: Porta’s index of heart rate asymmetry
HRA: Heart rate asymmetry
HRV: Heart rate variability
PRSA: Phase rectified signal averaging
RRI: R-R interval
T_I_: Inspiratory duration
T_E_: Expiratory duration
