# Supplementary material for: Different effects of inspiratory duration and expiratory duration on heart rate deceleration capacity and heart rate asymmetry
Source: Eur J Appl Physiol. 2024 Feb 29;124(7):2101–10. doi: 10.1007/s00421-024-05433-2 (PMC11199285; doi:10.1007/s00421-024-05433-2)
Supplement: Supplementary file 1 — Supplementary file1 (DOCX 26 KB) [file 421_2024_5433_MOESM1_ESM.docx]

**Different effects of inspiratory duration and expiratory duration on heart rate deceleration capacity and heart rate asymmetry**

**Supplementary Information**

Yong-Ping Wang^1^, Terry B. J. Kuo^2,3,4,5,6^, Guo-Zhi Wang^7^, Cheryl C. H. Yang^2,3,4,5*^

^1^Department of Anesthesiology, National Taiwan University Hospital, Taipei, Taiwan.

^2^Institute of Brain Science, National Yang Ming Chiao Tung University, Taipei, Taiwan.

^3^Sleep Research Center, National Yang Ming Chiao Tung University, Taipei, Taiwan.

^4^Brain Research Center, National Yang Ming Chiao Tung University, Taipei, Taiwan.

^5^Department of Education and Research, Taipei City Hospital, Taipei, Taiwan.

^6^Clinical Research Center, Taoyuan Psychiatric Center, Ministry of Health and Welfare, Taoyuan, Taiwan.

^7^Department of Surgery, Chung Shan Medical University Hospital, Taichung, Taiwan.

*Corresponding author

**Supplementary Table S1.** Actual respiratory phase durations during paced breathing

|  | Inspiratory setting | I2 | I4 | I6 |
| --- | --- | --- | --- | --- |
| Expiratory setting |  |  |  |  |
| E2 | Average inspiratory duration (s) | 1.94 ± 0.05 | 3.81 ± 0.14^a^ | 5.85 ± 0.21^a,b^ |
|  | SD of inspiratory duration (s) | 0.16 ± 0.05 | 0.25 ± 0.07^a^ | 0.33 ± 0.19^a^ |
|  | Average expiratory duration (s) | 2.06 ± 0.05 | 2.19 ± 0.15 | 2.16 ± 0.22^a^ |
|  | SD of expiratory duration (s) | 0.19 ± 0.05 | 0.28 ± 0.07^a^ | 0.33 ± 0.15^a^ |
| E4 | Average inspiratory duration (s) | 1.91 ± 0.11 | 3.83 ± 0.18^a^ | 5.75 ± 0.23^a,b^ |
|  | SD of inspiratory duration (s) | 0.20 ± 0.08 | 0.29 ± 0.15^a^ | 0.36 ± 0.21^a^ |
|  | Average expiratory duration (s) | 4.10 ± 0.11^c^ | 4.17 ± 0.19^c^ | 4.25 ± 0.23^a,c^ |
|  | SD of expiratory duration (s) | 0.27 ± 0.10 | 0.32 ± 0.13^a^ | 0.38 ± 0.20^a^ |
| E6 | Average inspiratory duration (s) | 1.99 ± 0.12 | 3.91 ± 0.25^a^ | 5.85 ± 0.18^a,b^ |
|  | SD of inspiratory duration (s) | 0.21 ± 0.06 | 0.27 ± 0.08^a^ | 0.31 ± 0.11^a^ |
|  | Average expiratory duration (s) | 6.00 ± 0.12^c,d^ | 6.09 ± 0.25^c,d^ | 6.16 ± 0.17^a,c,d^ |
|  | SD of expiratory duration (s) | 0.31 ± 0.14^c^ | 0.36 ± 0.15^a,c^ | 0.36 ± 0.11^a,c^ |

SD, standard deviation. Values are presented as mean ± SD. Two-way repeated-measures analysis of variance was performed to analyze differences among various settings for inspiration (I2, I4, and I6) and expiration (E2, E4, and E6) durations. Tukey’s test was used for multiple comparisons. No significant interactions were observed in all parameters. Main effects are shown. Values different from I2, I4, E2, and E4 are indicated using superscript letters a, b, c, and d, respectively.

**Supplementary Table S2.** F and *P* values in the two-way repeated-measures analysis of variance

|  |  | Inspiratory duration | | Expiratory duration | | Interaction | |
| --- | --- | --- | --- | --- | --- | --- | --- |
|  |  | F | *P* | F | *P* | F | *P* |
| Fig. 5 | Deceleration Capacity | 7.87 | 0.002 | 12.46 | <0.001 | 1.03 | 0.40 |
|  | Acceleration Capacity | 0.38 | 0.69 | 9.12 | <0.001 | 3.57 | 0.01 |
|  | Difference in magnitude | 8.60 | 0.001 | 9.22 | <0.001 | 1.29 | 0.29 |
|  | HRA_DC_ | 10.61 | <0.001 | 10.02 | <0.001 | 0.92 | 0.46 |
| Fig. 6 | HRA_GI_ | 8.02 | 0.002 | 11.72 | <0.001 | 2.94 | 0.03 |
|  | HRA_PI_ | 10.62 | <0.001 | 10.68 | <0.001 | 0.76 | 0.56 |
